# 5q Deletion Myelodysplastic Syndrome in a Young Male Patient

**DOI:** 10.7759/cureus.17466

**Published:** 2021-08-26

**Authors:** Sherif Elkattawy, Sarah Ayad, Iman El-Feki, Xutong Guo, Edmund Appiah-Kubi, Afrah Talpur, William Kessler

**Affiliations:** 1 Internal Medicine, Rutgers-New Jersey Medical School/Trinitas Regional Medical Center, Elizabeth, USA; 2 Internal Medicine, St. George's University, West Indies, GRD; 3 Hematology and Medical Oncology, Rutgers-New Jersey Medical School/Trinitas Regional Medical Center, Elizabeth, USA

**Keywords:** myelodysplastic syndrome, lenalidomide, 5q deletion, anemia, thrombocytopenia.

## Abstract

Myelodysplastic syndromes (MDS) are a diverse group of hematopoietic stem cell malignancies with various phenotypic variability that are categorized by abnormal differentiation of one or multiple cell lines of the bone marrow. A large part of the phenotypic heterogeneity is in part due to the wide set of genetic defects related to MDS. Though clinically, MDS is centered on diagnostic measures that do not incorporate molecular genetic data, an isolated deletion of the long arm of chromosome 5 (del(5q)) is the only subset of MDS to be identified by genetic defects. This distinctive phenotype is termed 5q-syndrome. We report a case of a 25-year-old with a past medical history of polydactyly, severe anemia, and thrombocytopenia who presented to the emergency department with a chief complaint of weakness and fatigue. Bone marrow biopsy showed myeloid neoplasm with complex genetic abnormalities, nearly 100% hyperplastic marrow with marked trilineage dysplasia, relative myeloid hyperplasia with increased abnormal eosinophilic precursors, erythroid left shift, and atypical megakaryocytes. Fluorescence in situ hybridization (FISH) panel showed deletion of 5q-. Herein, we address the clinical course and morphological characteristics as well as possible therapeutic options for 5q syndrome.

## Introduction

Myelodysplastic syndrome (MDS) is a stem cell disorder characterized by ineffective hematopoiesis due to defects in differentiation of bone marrow cell lineages [1]. This syndrome increases the amount of immature cells with a reduced quantity of mature blood cells thereby increasing the occurrence of anemia, thrombocytopenia, and neutropenia [2]. MDS can be caused by environmental factors (radiation, benzene, chemotherapy) and genetic factors. The most predominant genetic cause in 50% of patients with MDS is due to an isolated deletion of the long arm of chromosome 5 (del(5q)). The deleted region contains 40 genes which are important for blood cell development. Damage to 5q31 and 5q33 regions leads to haploinsufficiency of ribosomal protein S14 gene (RPS14 gene). The loss of RSP14 increases levels of p53 primarily in erythroblasts, promotes their apoptosis, and results in a differentiation defect [1,3]. The loss of microRNA-145 (miR-145) or microRNA-146a (miR-146a) gene contributes to megakaryocyte and platelet abnormalities [1]. Patients with del(5q) MDS have a favorable prognosis with a survival rate of 58 months and a low risk of progression to acute myeloid leukemia (AML). Lenalidomide is the drug of choice with a 67% remission rate in patients [4].

## Case presentation

Our patient is a 25-year-old, relatively short stature Hispanic male with a past medical history of polydactyly, severe anemia, thrombocytopenia, recurrent oral infections from Guatemala who presented to the emergency department (ED) with a chief complaint of a one-month duration of progressively worsening weakness and fatigue. He had poor exercise tolerance, dizziness, multiple recurrent near syncopal episodes, and one unwitnessed syncopal episode. The patient also endorsed weight loss for the past few months; however, he could not quantify it. On review of systems, the patient denied tongue biting, urine incontinence, fever, nausea, vomiting or diarrhea, black stools, blood in urine, or epistaxis. Social history was significant for chronic alcohol consumption. He denied any family history of cancer or bleeding disorders. 

The patient was awake, alert, oriented, and hemodynamically stable upon ED admission. He was afebrile, borderline tachycardia at 101 beats per minute, respiration rate 20 breaths/min, O2 saturation was 100% on room air, and blood pressure was 107/68 mmHg. The physical exam showed short stature with normocephalic/atraumatic, possible microcephaly with pupil equal, round, reactive to light and accommodation, extraocular movement intact, slight icteric sclera, widely spaced teeth, irregular ridged ovoid tongue. The neck was supple, no lymphadenopathy, and jugular venous pulsations (JVP) of 6-8 cm. Lungs clear to auscultation bilaterally. The cardiac exam showed regular rate rhythm with S1, S2 heard, no murmur, rub, and gallops. The abdomen was soft, non-tender, non-distended, normal active bowel sounds; no organomegaly or masses were noted. The extremities exam was significant for black streak and vertical nail ridges on all four extremities. Skin exam showed petechiae of palms and soles. Whitish spots throughout the body and blackish purple papules in groups were observed on bilateral arms.

Laboratory test results were as follows: normal white blood cell count: 8.0 K/uL, differential count (polymorphous leukocyte 72%, slightly low lymphocytes at 16%, monocytes 7%, basophils 2%, minimally increased metamyelocytes at 1%), severe anemia with RBC 1.09 M/uL, hemoglobin at 3.8 GM/dL, reticulocyte 6.8%, hematocrit 12.2%, mean corpuscular volume (MCV) 111.9 FL, mean corpuscular hemoglobin (MCH) 34.4 PG, mean corpuscular hemoglobin concentration (MCHC) 30.7 G/dL, red cell distribution width (RDW) 20.3%, platelet 54K/uL, mean platelet volume (MPV) 11.3 FL, glucose level 134 mg/dL, slightly low calcium 8.5 mg/dL, elevated urobilinogen 4.0 E.U/dL, slightly elevated prothrombin time (PT) and activated partial thromboplastin time (APTT) at 15.0 sec and 22.8 sec respectively, elevated alanine aminotransferase (ALT) at 75 U/L, lactate dehydrogenase 478. Blood work did not show any vitamin deficiencies nor malnutrition.

The patient had a low absolute reticulocyte count at 2.0 and reticulocyte index at 0.79, which indicated hypo-proliferation. Hematology-oncology was consulted, who reviewed the peripheral smear that showed nucleated RBC and teardrop cells. Bone marrow biopsy was recommended, and specimens were drawn from the right posterior iliac bone. The biopsy showed myeloid neoplasm with complex genetic abnormalities, nearly 100% hyperplastic marrow with marked trilineage dysplasia, relative myeloid hyperplasia with increased abnormal eosinophilic precursors, erythroid left shift, atypical megakaryocytes, no increase in blasts or mast cell proliferation, mild reticulin fibrosis. Given the biopsy findings, a defined diagnosis could not be obtained, and cytogenic analysis, flow cytometry, and karyotype were recommended. The laboratory results are discussed in (Tables 1-3). 

**Table 1:** Myelodysplasia, AML, eosinophilia FISH panel FISH: fluorescence in situ hybridization; AML: acute myeloid leukemia; RARA: retinoic acid receptor alpha; NF1: neurofibromatosis type 1; MLL: mixed-lineage leukemia; PML: promyelocytic leukemia; CBFB: core binding factor beta; PDGFRf: platelet-derived growth factor-receptor f; PDGFRa: platelet-derived growth factor receptor a; FGFR1: fibroblast growth factor receptor 1.

FISH panel	Interpretation
Myelodysplasia FISH panel	Deletion of 5q- 3 copies RARA (17q21) 3 copies NF1 (17q11.2) 3 copies 5q15. Partial deletion of 5’ region of MLL
Acute myeloid leukemia FISH panel	No BCR/ABL1, PML/RARA, ETO/AML1, CBFB detected.
Eosinophilia FISH panel	One copy of PDGFRf (5q32) is detected. No FGFR1, PDGFRa, PDGFRf detected

**Table 2:** Flow cytometry results

Flow Cytometry Results
Clonal hematopoiesis with marked left shift. Myeloid hyperplasia, dysplasia, atypical eosinophils and 2% (CD34+) blasts.

**Table 3:** Cytogenetic karyotype analysis result

Karyotype result	46, XY, pseudo dicentric (3;17) (q25; p11.2), derivative chromosome (8) t (5;8) (p11.2;p11.2), add (10) (q26) (13) /46, idem, del (5)| (q22q33) (8)

The bone marrow biopsy does not suggest any form of AML. It showed hypercellularity with some erythroid dysplasia on the smear. As a result, DiGuglielmo’s syndrome was suspected. While the patient is young for MDS, based on the FISH panel, flow cytometry, and karyotype, MDS was suspected. The patient had been managed with necessary blood transfusion, fresh frozen plasma, and continuous monitoring for hemoglobin and platelet status. In light of short stature, microcephaly, suspected cognitive deficiency, and another congenital disorder was suspected. As a result, the patient was transferred to another hematology facility for further evaluation. 

## Discussion

MDS is a rare blood cancer disorder that is characterized by ineffective hematopoiesis. Several subtypes have been identified, of which 5q deletion is the most common and accounts for 10%-20% of all MDS [5]. Upregulation of the p53 pathway in MDS del(5q) subtype was studied by Gaballa and Besa and it was demonstrated that this deletion affects regions 5q31 and 5q33 that lead to p53 activation [3]. This causes a decrease in erythropoiesis, as a result of accumulation of p21 in erythroid precursor cells [6], which triggers apoptosis in a p53-dependent manner.

Patients typically present with macrocytic anemia, normal or high platelet count, hypolobulated megakaryocytes, and reduced risk of transformation to AML [6]. Their clinical presentation is mainly attributed to the effects caused by cytopenia and thus, patients have symptoms of anemia such as fatigue, possible bleeding complications, and infections which are more evident later on in the disease course [7]. The hallmark feature of MDS is the morphological dysplasia of premature and mature bone marrow blood cells, which was evident in our patient who presented with atypical megakaryocytes and relative myeloid hyperplasia. In particular, this description fits the spectrum of DiGuglielmo’s syndrome, which is a spectrum of disorders that involves abnormal erythroid precursor hyperplasia and abnormal sideroblasts [8]. This syndrome primarily involves erythroid cells of the bone marrow, as described by Giovanni Di Guglielmo in the 1920s, but later it became apparent that the erythroleukemia can be mixed involving both erythroblastic and myeloblastic forms [9].

MDS is a complex disease that poses difficulties in diagnosis and management as targeted treatments are limited. Management is shifting towards taking a personalized approach and utilizing risk stratification to assesses the patient’s severity of cytopenia, serum erythropoietin (EPO) levels, manifestation of 5q deletion, percent of blasts in the bone marrow, age, and comorbidities to determine treatment outcome [7,10]. However, supportive care is the basis of therapy, which includes treating symptomatic anemia with RBC transfusions and severe thrombocytopenia with platelet transfusions [10]. Recent studies have shown that the quality of life is not necessarily increased with higher transfusion targets [7] and thus, transfusion therapy should largely be determined by the patient’s subjective symptoms. Infection is the leading cause of death in this patient population and as a result, a spike in fever should be promptly investigated. In particular, patients with MDS del(5q) who are taking lenalidomide, a drug with great sensitivity against haploinsufficient del(5q) cells, are at higher risk of neutropenia, and adding granulocyte-colony stimulating factor (G-CSF) has been recommended when their absolute neutrophil count (ANC) falls below 1.0x109/L [11]. Nevertheless, lenalidomide has been shown to be effective in MDS as it is involved in inducing p53 degradation [6] which helps to restore normal functions of mouse double minute 2 homolog (MDM2), a protein that can reduce the rate of apoptosis in erythroblast cell production. Although immunosuppressive agents have variable results in MDS, Dou and Fang have found that pre-treatment of lenalidomide before initiating cyclosporine helped negate immunosuppression of T cells that is normally created by cyclosporine [12]. Therefore, a combination of both therapies together could be explored to help create better outcomes for these patients. Other targeted treatments that have been studied include azacytidine and decitabine, which are hypomethylating agents, that showed improved overall survival and response rate [7] and can be used in patients with response failure to lenalidomide [13].

## Conclusions

MDS is a rare blood cancer disorder that is characterized by ineffective hematopoiesis. An isolated deletion of the long arm of chromosome 5 (del(5q)) is a subset of MDS. MDS is a complex disease that poses difficulties in diagnosis and management as targeted treatments are limited. However, lenalidomide is the drug of choice with a 67% remission rate in patients. Herein, we present a case of an MDS 5q deletion in our young male patient who presented with progressive fatigue, thrombocytopenia, and severe anemia. Patients with MDS and 5q deletion respond well to lenalidomide, thus an early diagnosis of these genetic abnormalities is crucial. 
